# miR-487b, miR-3963 and miR-6412 delay myogenic differentiation in mouse myoblast-derived C2C12 cells

**DOI:** 10.1186/s12860-015-0061-9

**Published:** 2015-04-30

**Authors:** Naoki Katase, Kumiko Terada, Takahiro Suzuki, Shin-ichiro Nishimatsu, Tsutomu Nohno

**Affiliations:** Department of Molecular and Developmental Biology, Kawasaki Medical School, 577 Matsushima, Kurashiki, Okayama 701-0192 Japan

**Keywords:** Microrna (miRNA), C2C12, myoblast-derived cell, Skeletal muscle, Myogenic differentiation, miR-487b, miR-3963, miR-6412

## Abstract

**Background:**

Skeletal muscle differentiation is a multistep, complex pathway in which several important signaling molecules are involved. Recently, microRNAs (miRNAs), endogenous non-coding small RNAs that regulate mRNAs, have been proposed to be involved in skeletal muscle differentiation. In this study, we identified skeletal muscle differentiation-associated miRNAs by comparing miRNA expression profiles between C2C12 cells and Wnt4 over-expressing C2C12 cells (W4-08), which can spontaneously differentiate into myotubes.

**Results:**

We identified miR-206, miR-133a, and miR-133b as up-regulated miRNAs and miR-487b, miR-3963 and miR-6412 as down-regulated miRNAs in differentiating cells. We focused on the down-regulated miRNAs because their functions were largely unknown. Transfection of mimics of these miRNAs into C2C12 cells resulted in significantly reduced expression of myogenic differentiation markers, including troponin T and myosin heavy chain fast type and slow type, but did not affect the expression of the myogenic transcription factors, MyoD and myogenin.

**Conclusions:**

These miRNAs were characterized as new myogenic differentiation-associated miRNAs which may delay late myogenic differentiation or maturation.

**Electronic supplementary material:**

The online version of this article (doi:10.1186/s12860-015-0061-9) contains supplementary material, which is available to authorized users.

## Background

Skeletal muscle development is complex, involving several signaling pathways. Skeletal muscles are formed from somites, which are derived from paraxial mesoderm. Somites form initially as somitomeres, and after the epithelization process, they form mesenchymal sclerotome and dermomyotome. Mesodermal cells are selected to form myoblasts and subsequently differentiate into myotubes [1]. The molecular mechanism determining myogenesis was recently identified. The Wnt protein plays crucial roles in the development of skeletal muscle. Indeed, skeletal muscle is formed from paraxial mesoderm under the influence of signals from the neural tube and dorsal ectoderm, including Wnt proteins and BMP4 signaling, and myogenic cell fate is determined by intrinsic factors, MyoD and Myf5 [2-5]. The diverse Wnt members function differently. For example, Wnt1 expression preferentially activates Myf5, while Wnt7a-expressing cells activate MyoD, and Wnt4, Wnt5a and Wnt6 can activate both Myf5 and MyoD [6]. Wnt1, Wnt 3a and Wnt 5a induce the proliferation of satellite cells, a source of myoblasts [7].

Wnt4 over-expression in mouse myoblast-derived C2C12 cells induces hypertrophy of myotubes. Wnt4 over-expression in the presumptive limb field of the chick embryo results in up-regulation of PAX7 and MyoD, and increased skeletal muscle mass. Wnt4 over-expression in C2C12 cells induces an increase in fast-type myosin heavy chain (MyHC) expression. Moreover, Wnt4 is reported to negatively regulate myostatin [8,9]. Therefore Wnt4 is thought to possess myogenic activity.

Previously, we generated a Wnt4-over-expressing cell line from C2C12 cells, W4-08 [4]. C2C12 cells can differentiate into myotubes under reduced mitogen conditions, while W4-08 cells can spontaneously differentiate into myotubes even in proliferative conditions. We investigated the factors that specifically affect myogenic differentiation by comparing this Wnt4-over-expressing cell line with its parental C2C12 cell line.

MicroRNAs (miRNAs) are small, endogenous non-coding RNAs consisting of approximately 22 nucleotides that regulate mRNA expression by binding to complementary sequences of the mRNA leading to cleavage or translational repression [10]. These small single strand miRNAs target one or more mRNAs and play important roles in cell embryogenesis, cell differentiation, carcinogenesis and apoptosis [11]. Recently, involvement of miRNAs in skeletal muscle differentiation has been indicated [12].

Therefore, we searched for specific miRNAs that are involved in myogenic differentiation, and our miRNA microarray data demonstrated two novel miRNAs, miR-487b, miR-3963 and miR-6412 that are specifically down-regulated in C2C12 cells cultured in differentiation medium.

Transfection of the mimics of these miRNAs resulted in decreased expression of the myogenic markers, troponin T and myosin heavy chain (MyHC), suggesting delayed myogenic differentiation. Our results suggest that miR-487b, miR-3963 and miR-6412 are novel muscle-specific miRNAs that may function as negative regulators of skeletal muscle differentiation.

## Results and discussion

### miRNA microarray analysis

We compared the miRNA expression profiles of C2C12-PR cells (C2C12 cells grown in proliferation medium) vs. W4-08 cells (Wnt4-over-expressing C2C12 cells) cultured in proliferation medium. W4-08 cells can differentiate into myotubes even in proliferative conditions. We identified 161 miRNAs to be up-regulated, including miR-206, miR-133b and miR-133a (by 5.84, 5.46 and 5.37 fold in log2 ratio, respectively), while only miR-487b, miR-6412 and miR-3963 were down-regulated by about −1.00 in log2 ratio (−1.70, −0.96 and −0.93 fold in log2 ratio, respectively). The expression of these up- and down-regulated miRNAs were also commonly altered when expression in C2C12-PR vs. C2C12-DF cells (C2C12 cells grown in differentiation medium) was compared; 42 miRNAs were up-regulated with miR-206, miR-133b and miR-133a being commonly up-regulated (2.21, 1.68 and 1.63 fold in log2 ratio, respectively), and miR-487b, miR-6412 and miR-3963 were down-regulated (−1.98, −2.99 and −2.13 fold in log 2 ratio, respectively). (see Gene Expression Omnibus (GEO) at NCBI with accession number GSE63454.)

Therefore, we focused on miR-206, miR-133a, miR-133b, miR-487b, miR-6412 and miR-3963 as a potential myogenic differentiation-related miRNAs, and the effect of transfection of these miRNAs on cell differentiation was assessed.

### The effect of miRNA transfection on C2C12 differentiation

**Figure 1:**
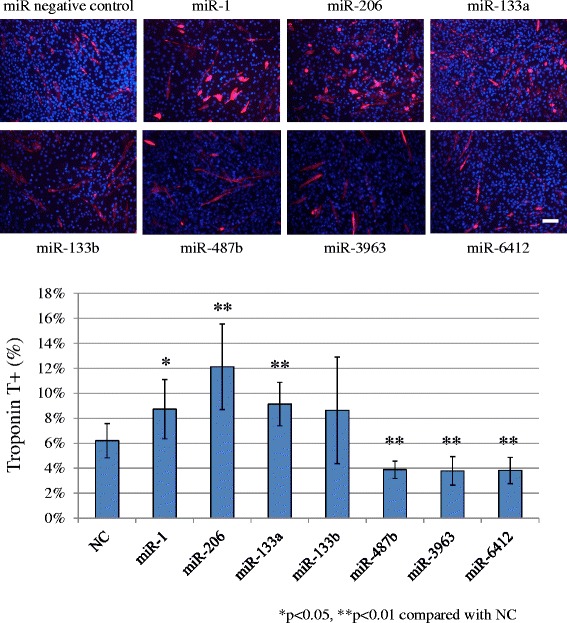
Troponin T expression, 3 days after transfection and medium change. Compared with the miR negative control (NC), transfection of miR-1, miR-206 and 133a resulted in a significantly higher troponin T positive ratio, while miR-487b, miR-3963 and miR-6412 transfectants gave a significantly lower ratio. Scale bar 50 μm. NC: negative control.

We transfected miRNA mimics of the miRNAs which were significantly up- or down-regulated, and effects on cellular differentiation were determined by immunocytochemistry. Transfection efficacy of miRNA mimics was confirmed by real time RT-PCR analysis of PTK9 expression following miR-1 positive control transfection (Additional file: 1 Figure S1). Troponin T expression was evaluated after the transfection of the mimics followed by 3 days incubation in differentiation conditions. The troponin T expression ratio reached approximately 6% in negative controls. Transfection of miR-1 positive control, miR-206 and miR-133a resulted in a significant increase of the troponin T-positive cell ratio, while transfection of miR-487b, miR-3963 and miR-6412 mimics significantly decreased troponin T expression (Figure 1).

**Figure 2:**
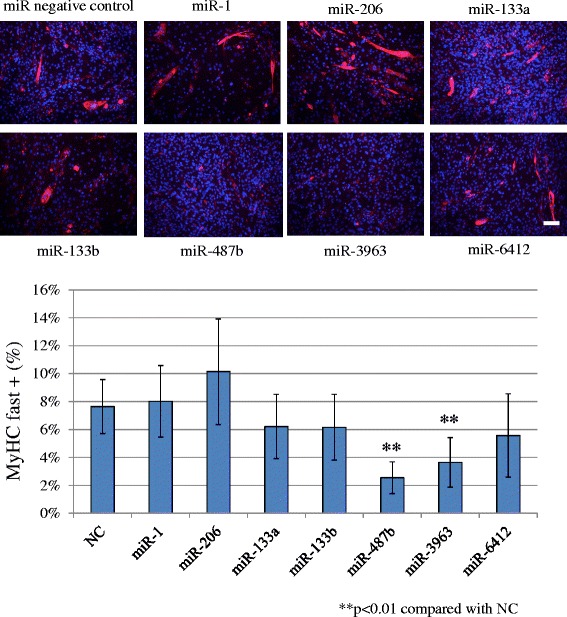
miR-487b and miR-3963 transfection down-regulated fast type MyHC. miR-1, miR-206, miR-133a, miR-133b, miR-6412 did not affect MyHC expression. Scale bar 50 μm. NC: negative control.

**Figure 3:**
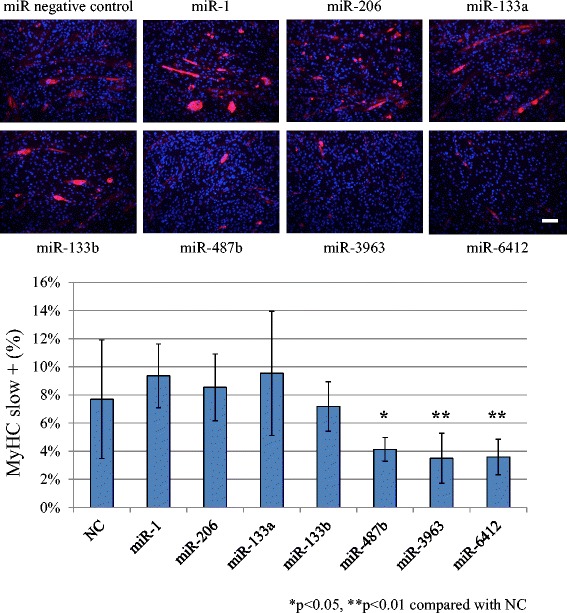
miR-487b, miR-3963 and miR-6412 transfection down-regulated slow type MyHC. miR-1, miR-206, miR-133a and miR-133b did not affect MyHC expression. Scale bar 50 μm. NC: negative control.

Cellular differentiation was also assessed by determining levels of fast and slow type MyHCs. Immunocytochemistry for MyHCs was performed after transfection followed by 1 week of incubation in differentiation medium. Transfection of miR-487b and miR-3963 mimics resulted in a significant decrease in the MyHC (fast) positive ratio (Figure 2), while miR-1 positive control, miR-206, miR-133a, miR-133b and miR-6412 did not affect the MyHC (fast) positive cell ratio. miR-487b, miR-3963 and miR-6412 mimics significantly decreased the MyHC (slow) positive cell ratio (Figure 3).

### miR-487b, miR-3963 and miR-6412 significantly suppress expression of troponin T

**Figure 4:**
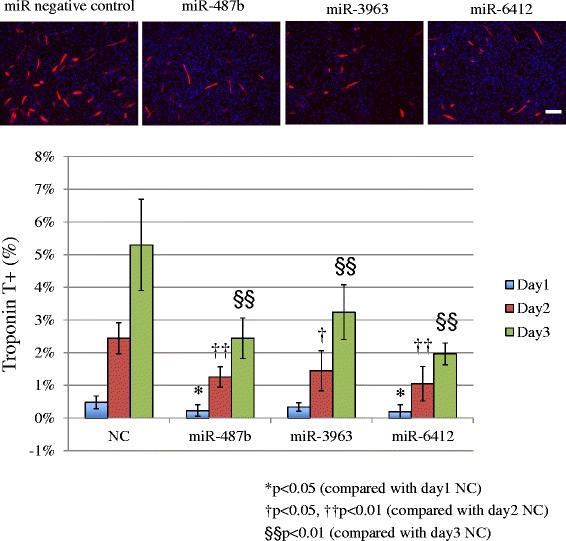
Troponin T expression from day 1 to day 3 after transfection and medium change. Troponin T expression increased on successive days. The miRNA mimic transfection group showed a lower troponin T expression ratio. miR-487b and miR-6412 transfection resulted in significantly lower troponin T expression from day 1. Scale bar 50 μm. NC: negative control.

Next, we focused on miR-487b, miR-3963 and miR-6412 transfection, which resulted in decreased differentiation. We confirmed that the expression of these miRNAs was significantly decreased in differentiation conditions by RT-qPCR (Additional file: 2 Figure S2). The expression of these three miRNAs was significantly down-regulated in C2C12 cells cultured in differentiation medium, compared with cells cultured in proliferation medium. The mimics of these miRNAs were transfected in C2C12 cells. To confirm when the reduction of troponin T expression begins, its expression was assessed on days 1, 2 and 3 after transfection. The troponin T expression ratio increased on each day in all groups, and transfection of miRNA mimics suppressed its expression significantly (Figure 4). The suppression of troponin T expression was observed from day 1 for miR-487b and miR-6412, and from day 2 for miR-3963. Next, to address the possibility that these miRNAs might suppress an earlier myogenic differentiation stage, we assessed the expression of myogenic transcription factors, MyoD and myogenin.

### miRNA mimic transfection and MyoD and myogenin expression in C2C12 cell

**Figure 5:**
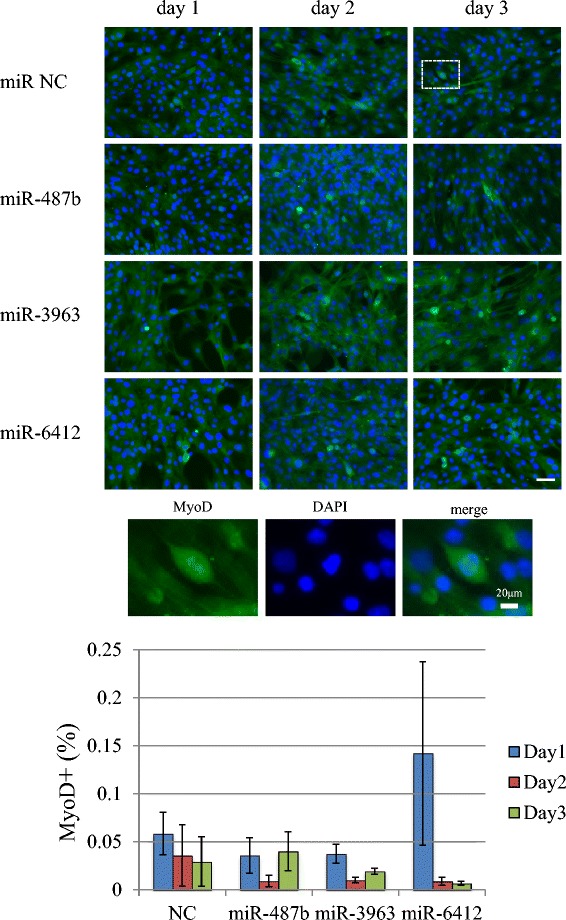
MyoD expression in miRNA transfectants from day 1 to day 3 after transfection and medium change. The nuclear staining pattern is shown (Green: Myo D, Blue: DAPI, photos were taken from the area indicated in the white rectangular). MyoD expression was observed in both the cytoplasm and nucleus. Scale bar 50 μm. MyoD nuclear staining was counted and is presented in the bar graph. There were no significant differences in MyoD nuclear positive ratios. NC: negative control.

**Figure 6:**
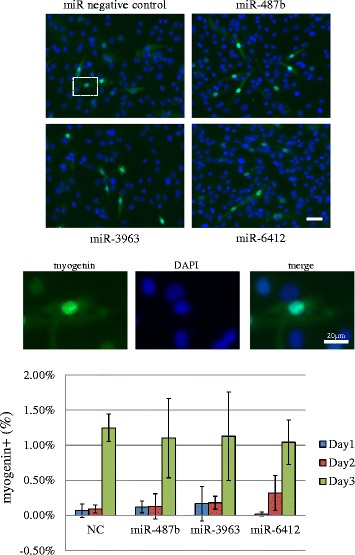
The expression of myogenin in miRNA transfectants on day 3 after transfection. Expression of myogenin was observed in the cytoplasm and nucleus. The nuclear staining pattern is shown (Green: myogenin, Blue: DAPI, photos were taken from the area indicated in the white rectangular). Nuclear myogenin expression was counted. There were no significant differences in myogenin nuclear positive ratios. Scale bar 50 μm. NC: negative control.

MyoD expression was assessed on day 1, 2 and 3 after transfection. MyoD and myogenin expression was observed from day 1–3 in the nucleus. A positive reaction in the nucleus is presented as the ratio of cells showing a positive nuclear reaction. The nuclear expression ratio of MyoD showed varied scores, but there was no significant difference among miR negative control and miRNA mimic transfection groups (Figure 5). The myogenin nuclear expression ratio increased on each day, but transfection of miRNA mimics did not significantly affect its expression pattern (Figure 6). We do not think that MyoD and myogenin cellular localization is altered by transfection of miRNA mimics because we confirmed that the expression patterns of these molecules in non-transfected C2C12 cells were similar to those of the miRNA transfection group (data not shown).

Skeletal muscle differentiation is a multistep pathway in which several signaling molecules are implicated. In particular, Wnt signaling plays important roles in myogenic commitment and differentiation. The formation of skeletal muscle from paraxial mesoderm is influenced by signals from the neural tube and dorsal ectoderm, including Wnt4 and BMP4 [4]. And in adult skeletal muscle, several Wnt members including Wnt1, Wnt3, Wnt5a and Wnt7a are required for proliferation and differentiation of satellite cells during myotube turnover [5,7]. Previously we established the W4-08 cell line, which stably over-expresses Wnt4 and spontaneously differentiates into myotubes even in mitogen rich medium. Comparison of gene expression profiles between W4-08 cells and its parental cell line, C2C12, may be informative for the understanding of myogenesis-related molecules. Recently, involvement of miRNAs in skeletal muscle differentiation has been debated. For example, miR-1, miR-206, miR-133 [13,14] are known as muscle-specific miRNAs. Additionally, miR-23a [15], miR-24 [16], miR-26 [17], miR-27a [18,19], miR-27b [20], miR-29 [21], miR-124 [22], miR-128a [23], miR-146b [24], miR-148a [25], miR-155 [26], miR-181 [27], miR-199 [28], miR-186 [29], miR-214 [30], miR-221/222 [31], miR-351 [32], miR-486 [33], miR-489 [34], miR-499 [35] and miR-3906 [36] are reported to be involved in skeletal myogenesis. miRNAs can either inhibit or promote myogenesis, depending on their target mRNAs.

We found that miR-206, miR-133a, miR-133b were up-regulated, and miR-487b, miR-3963, and miR-6412 were down-regulated during myogenic differentiation. Consistent with our previous report, transfection of miR-206, and miR-133a resulted in significantly higher troponin T expression. However, miR-206, 133a and 133b did not affect the expression of slow type or fast type MyHC. We used miR-1 as a positive control, which was as assayed by the down-regulation of the PTK9 target gene (Additional file: 1 Figure S1). Transfection of miR-1 also showed significantly higher troponin T expression because miR-1 also regulates proliferation and differentiation of myoblast cells by targeting the transcription of HDAC4 [14]. However, these miRNAs, which were significantly up-regulated in W4-08 cells, did not affect slow type or fast type MyHC, suggesting that these miRNAs are not significantly associated with the late muscle differentiation step.

In this study, we identified miR-486b, miR-3963 and miR-6412 as novel myogenic differentiation-related miRNAs, the functions of which are largely unknown. Transfection of these miRNAs resulted in reduced expression of myogenic differentiation markers, particularly in the late myogenic differentiation stage. However, involvement of these miRNAs in myogenic commitment or differentiation has not been reported. Moreover, no reports have been published on the function of miR-3963 or miR-6412. Some reports suggested that miR-487b is associated with several kinds of pathological condition. miR-487b is commonly and severely down-regulated in prostate cancer cell lines, suggesting that miR-487b may function as a tumor suppressor that regulates cell proliferation, apoptosis, migration and invasion [37]. Furthermore, miR-487b expression is significantly associated with disease-free survival of non-MYCN-amplified favorable neuroblastoma [38,39]. miR-487b also promotes epithelial-mesenchymal transition in lung adenocarcinoma cell [40], while several other reports imply roles of miR-487b in cellular proliferation. miR-487b is predicted to target insulin receptor-like substrate, down-regulation of which may impair skeletal muscle growth [41,42]. Xi et al reported that miR-487b directly targets Wnt5a [43], which is important in proliferation of satellite cells in adult skeletal muscle.

We also sought the target of these novel myogenic differentiation-related miRNAs using a bioinformatics approach. The candidate target for miR-487b varied greatly depending on the database queried and it was difficult to determine a commonly suggested target. However, according to microRNASeq (https://cm.jefferson.edu/rna22v1.0/), miR-487b directly targets several molecules that are involved in skeletal muscle differentiation, including Wnt3, Wnt5a, troponin T type 3 (skeletal, fast) (TNNT3), myosin, heavy chain 1, skeletal muscle, adult (MYH1), myosin, heavy chain 2, skeletal muscle, adult (MYH2), myosin, heavy chain 3, skeletal muscle, embryonic (MYH3) and myosin, heavy chain 4, skeletal muscle (MYH4). However, we were unable to identify candidate targets associated with muscle differentiation for miR-3963 and miR-6412.

These data indicate that these newly identified miRNAs may directly and/or indirectly suppress skeletal muscle differentiation and maturation.

## Conclusions

In the present study, we identified miR-487b, miR-3963 and miR-6412 as new skeletal muscle differentiation-associated miRNAs. Transfection of mimics of these miRNAs resulted in reduced expression in troponin T and MyHC, but did not affect the expression of MyoD or myogenin. These miRNAs may delay skeletal muscle differentiation by suppressing myotube differentiation and maturation markers. Further investigation is warranted to identify the targets of these miRNAs and the detailed mechanism by which they inhibit skeletal muscle maturation.

## Methods

### Over-expression of Wnt4 in C2C12 cells

The C2C12 cell line (a myoblast-like cell line from the C3H mouse) was purchased from the RIKEN Bioresource Center (Tsukuba, Japan) and maintained in Dulbecco’s modified Eagle’s medium (DMEM) supplemented with 10% fetal bovine serum (FBS) (Nichirei, Tokyo, Japan).

Stable *Wnt4* transfectants were established as previously described [4,44]. Briefly, After 12 to 24 h of subculture, an expression vector bearing a V5-tagged *Wnt4* cDNA (P_CMV_-Wnt4-V5-P_PGK_-blasticidin-SV40pA) was transfected into C2C12 cells. Transfected cells were then cultured in DMEM containing 10% FBS and blasticidin (Life Technologies, Carlsbad, CA, USA). After serial passages in blasticidin containing selection medium for 4 to 5 weeks, *Wnt4*-expressing stable transfectants were obtained.

The stable *Wnt4*-expressing C2C12 cells and parental cells were cultured in either proliferation medium (PR) containing 10% FBS in DMEM or in differentiation medium (DF) consisting of 2% horse serum (Sigma-Aldrich, St. Louis, MO, USA) in DMEM.

### miRNA expression analysis

miRNA expression analysis was performed using total RNA extracted from parental and *Wnt4-*expressing C2C12 cells under proliferation or differentiation conditions.

Total RNA was extracted from cultured cells using Isogen (NIPPON GENE, Toyama, Japan) according to the manufacturer’s instructions. Expression profiles were examined under the following conditions: C2C12 in PR vs. C2C12 in DF, C2C12 in PR and Wnt4-expressing C2C12 in PR, and C2C12 in DF and Wnt4-expressing C2C12 in PR.

Labeling, hybridization, scanning, and data processing were carried out with Toray 3D Gene (Toray, Tokyo, Japan). Minimum information about a microarray experiment-(MIAME-) compliant array data including raw data is deposited in the Gene Expression Omnibus (GEO) at NCBI with accession number GSE63454.

#### Transfection of miRNA

mirVana miR mimics used in this study were hsa-miR-206-3p (assay ID: MC10409), hsa-miR-133a-3p (MC10413), hsa-miR-133b-3p (MC10029), has-487b-3p (MC11296), mmu-miR-3963 (MC20952) and mmu-miR-6412 (MC26271) (Ambion, Life technologies). A scrambled miRNA, mirVana miRNA mimic negative control and a miR-1 positive control were used.

C2C12 cells were cultured in 100-mm diameter dishes until confluent, and transferred into 24-well plates in 2.5 × 10^4^ cells/ml/well. The next day, 16.5 pmol of miRNAs were transfected using Lipofectamine RNAiMAX (Invitrogen, Life Technologies) according to the manufacturer’s instructions. The next day, medium was changed to proliferation medium or differentiation medium and maintained for 1 day to 1 week.

#### Reverse transcription quantitative polymerase chain reaction (RT-qPCR)

The effect of miRNA transfection was determined by checking the expression of the PTK9 gene (a target of miR-1) using RT-qPCR [45]. The cells were harvested at 24 h, 48 h, 72 h and 1 week after medium change. Total RNA was extracted using Nucleospin RNA kits (MACHEREY-NAGEL GmbH, Düren, Germany). RNA was reverse transcribed into cDNA using ReverTra Ace (TOYOBO, Osaka, Japan) and used for quantitative PCR analysis.

The quantitative RT-PCR was performed using THUNDERBIRD qPCR mix (TOYOBO) and Step One Plus (Applied Biosystems, Life Technologies). The PCR conditions were 95°C for 1 min followed by 40 cycles of 95°C for 15 sec and 60°C for 45 sec.

For quantification, standards were prepared by the following procedures. RPS29 was chosen as a housekeeping gene [46], and the expression changes of PTK9 by the miR-1 positive control were evaluated as PTK9/RPS29. The sequences of the primers used are indicated in Table 1.

**Table 1:** **Primer sequences used in this study**

**mouse RPS29**	**Forward**	**5′-ATG GGT CAC CAG CAG CTC TA -3′**
	Reverse	5′- AGC CTA TGT CCT TCG CGT ACT -3′
mouse PTK9	Forward	5′- GAG AGC GGA TGC TGT ATT CC -3′
	Reverse	5′- CAG GAC CTT TCG GTT TAG CA -3′
M13	Forward	5′- GTA AAA CGA CGG CCA GT -3′
	Reverse	5 - CAG GAA ACA GCT ATG AC -3′
miR-487b	Forward	5′- AAG TGG ATG ACC CTG TAC GAT T - 3′
miR-3963	Forward	5′- TTG TGT CAG AAG TGG GAT ACA- 3′
miR-6412	Forward	5′- TAG TAG CTG AGG ATG GTT TCG A - 3′
miRNA Reverse		5′- GC GAG CAC AGA ATT AAT ACG AC -3′
Poly(T) Adaptor		5′- GCG AGC ACA GAA TTA ATA CGA CTC ACT ATA GGT TTT TTT TTT TTC G -3′

Standards were prepared as follows. Real-Time PCR was performed using the appropriate primers and the PCR products were separated by electrophoresis in 2% agarose gels. Then the bands were cut from the gel and amplicons were extracted using a QIAGEN Gel Extraction Kit (QIAGEN, Valencia, CA, USA). The PCR products were cloned with a pCRII-TOPO TA cloning kit (Life Technologies) into DH5α cells. Colonies were picked and cultured in LB medium, and plasmid DNA was extracted using a QiAprep Spin Miniprep kit (QIAGEN). After confirming correct sequences, plasmid inserts were amplified by PCR using M13 primers and Takara LA-Taq (Takara, Shiga, Japan) according to the manufacturer’s protocol. The PCR products were extracted using a MinElute PCR Purification Kit (QIAGEN) and then copy number was calculated. Finally, standards of 10^6^ to 10^2^ copies were prepared by serial dilution.

#### RT-qPCR for miRNA expression analysis

We performed quantitative PCR analysis to assess expression of miR-487b, miR-3963 and miR-6412. Total RNA samples were collected from C2C12 cells cultured in proliferation medium or differentiation medium. C2C12 cells were seeded on 100-mm dishes at 2.0 × 10^6^ cell/10 ml. The next day, media were changed to proliferation medium (DMEM containing 10% FBS) or differentiation medium (DMEM containing 2% HS). The cells were cultured for 3 days, and total RNA was extracted using ISOGEN (NIPPON GENE). cDNA synthesis and RT-qPCR was performed using Universal cDNA synthesis kit II (EXIQON, Woburn, MA, USA), ExiLENT SYBR® Green master mix (EXIQON) and MicroRNA LNA™ PCR primer set (EXIQON), according to the manufacturer’s protocols.

We also designed primers for miR-487b, miR-3963 and miR-6412 and performed quantitative PCR as reported previously [47]. The forward primers were designed based on the entire tested miRNA sequences, and the reverse primer was designed based on the poly (T) adaptor sequence. The sequences of all primers and the poly(T) adaptor are indicated in Table 1.

The cDNA was polyadenylated using a Poly(A) Tailing kit according to the manufacturer’s instructions (Ambion, Life technologies). After phenol-chloroform extraction and ethanol precipitation, the RNA was dissolved in nuclease free water, and reverse-transcribed using ReverTra Ace (TOYOBO) with 0.5 μg of poly(T) adaptor. RPS29 was selected as the internal reference gene for PCR quantification. Real-time PCR was performed using THUNDERBIRD qPCR mix (TOYOBO) and Step One Plus (Applied Biosystems, Life Technologies). The PCR conditions were 95°C for 10 min followed by 45 cycles of 95°C for 10 sec and 60°C for 1 min. The quantity of miRNAs, relative to the reference gene was calculated using the formula 2^-ΔCt^ (cycle threshold), where ΔCt = (Ct _miRNA_ –Ct _reference gene_). Comparison of miRNA expression was based on the ΔΔCt method [48,49], and the relative miRNA expression can be quantified according to the formula of 2^-ΔΔCt^, where ΔΔCt = (ΔCt of C2C12 in DF) − (ΔCt of C2C12 in PR).

### Immunocytochemistry

After transfection of miRNAs, cells were fixed in 4% paraformaldehyde for 10 min and rinsed with PBS three times. Then cells were treated for 30 min with blocking buffer, consisting of 3% BSA, 2% goat serum, 0.2% Tween 20 and 0.1% NaN_3_ in PBS. Then, cells were incubated with anti-troponin T (MAB1487, clone TT-98, Abnova, Taipei, Taiwan), anti-fast myosin heavy chain (MyHC) (MY-23, Sigma-Aldrich), anti-slow myosin heavy chain (NOQ7.5.4D, Sigma-Aldrich), anti-myogenin (F5D, Santa Cruz, Dallas, TX, USA) or anti-MyoD (C20, Santa Cruz) in blocking buffer at 4°C overnight. The dilution of antibodies was 1:200, 1:400, 1:1000, 1:50 and 1:50, respectively. After three TBS washes, cells were incubated with a 1:500 dilution of secondary Alexa Fluor 594-conjugated goat anti-rabbit or anti-mouse IgG antibody (Life Technologies) for 1 h. After three TBS washes, cell nuclei were stained with 1 μg/ml 4′,6′-diamino-2-phenylindole solution (DAPI, DOJINDO, Kumamoto, Japan). Fluorescent images were taken using an All-in-One Fluorescence Microscope BZ-9000 (Keyence Japan, Osaka, Japan). Total cell number and troponin T, MyHC (fast) or MyHC (slow), MyoD and myogenin-positive cells were counted and the results are presented as positive cell ratios. Approximately 1500-2000 cells/well in 6 wells were counted in each independent experiment, and each experiment was independently done more than three times to obtain the statistically significant results.

### Statistics

Significant differences between the control and the miRNA groups were determined using Student’s *t*-test. All computations were performed using PASW Statistics 18 (SPSS Inc., Chicago, IL, USA). P-value less than 0.05 was considered to indicate a statistically significant result.

## Additional files

Additional file 1: Figure S1. The efficacy of miRNA transfection was assessed by real time quantitative RT-PCR. miR-1 positive control transfection reduced its target gene (PTK9) expression significantly. RPS29 was used as a control housekeeping gene. Additional file 2: Figure S2. The expression of miR-487b, miR-3963 and miR-6412 was assessed by RT-qPCR. The expression of these three miRNAs was significantly down-regulated in C2C12 cells cultured in differentiation medium, compared with cells cultured in proliferation medium.
