# Erratum to: miR-487b, miR-3963 and miR-6412 delay myogenic differentiation in mouse myoblast-derived C2C12 cells

**DOI:** 10.1186/s12860-016-0083-y

**Published:** 2016-03-10

**Authors:** Naoki Katase, Kumiko Terada, Takahiro Suzuki, Shin-ichiro Nishimatsu, Tsutomu Nohno

**Affiliations:** Department of Molecular and Developmental Biology, Kawasaki Medical School, 577 Matsushima, Kurashiki, Okayama 701-0192 Japan

## Erratum

Following publication of the original version of this article [1] we noticed errors in Table 1 and Additional file 2: Figure S2 concerning the RT-qPCR data for comparison of miRNA expression between C2C12 cells grown in proliferation media (PR) and differentiation media (DF). The forward primer sequences for miR-487b, miR-3963 and miR-6412 should be identical to the sequences of miRNA themselves, whereas those used in the original version of the article were the reverse complementary sequences of these miRNAs. The forward primers in the original version correspond to the proportion of pre-miRNA of prospective miRNAs, which can form a hairpin structure and will be the source of mature miRNA.

**Table 1:** The sequence of the primers

Primer name		Sequence
mouse RPS29	Forward	5’-ATG GGT CAC CAG CAG CTC TA -3’
	Reverse	5’- AGC CTA TGT CCT TCG CGT ACT -3’
mouse PTK9	Forward	5’- GAG AGC GGA TGC TGT ATT CC -3’
	Reverse	5’- CAG GAC CTT TCG GTT TAG CA -3’
M13	Forward	5’- GTA AAA CGA CGG CCA GT -3’
	Reverse	5 - CAG GAA ACA GCT ATG AC -3’
miR-487b	Forward	5’- AAT CGT ACA GGG TCA TCC ACT T - 3’
miR-3963	Forward	5’- TGT ATC CCA CTT CTG ACA CA - 3’
miR-6412	Forward	5’- TCG AAA CCA TCC TCA GCT ACT A - 3’
miRNA Reverse		5’- GC GAG CAC AGA ATT AAT ACG AC -3’
Poly(T) Adaptor		5’- GCG AGC ACA GAA TTA ATA CGA CTC ACT ATA GGT TTT TTT TTT TTC G -3’

We re-designed correct primers for miR-487b, miR-3963 and miR-6412 and performed RT-qPCR again for confirmation. The results show that the expression level of these miRNAs significantly decreased in C2C12 cells grown in DF compared to those in PR.

The correct data and primer sequences are shown in the revised Table 1 and Figure S2. These changes do not change any other results or conclusions in the original version.

Additionally, during production of the original article an error was introduced into the email addresses of two of the authors. The correct email addresses are included in this article.

## Additional file

Additional file 2: Figure S2. (PPTX 62 kb)
