# Supplementary figures and images for: miR-487b, miR-3963 and miR-6412 delay myogenic differentiation in mouse myoblast-derived C2C12 cells
Source: BMC Cell Biol. 2015 Apr 30;16:13. doi: 10.1186/s12860-015-0061-9 (PMC4433089; doi:10.1186/s12860-015-0061-9)

## Slide 1
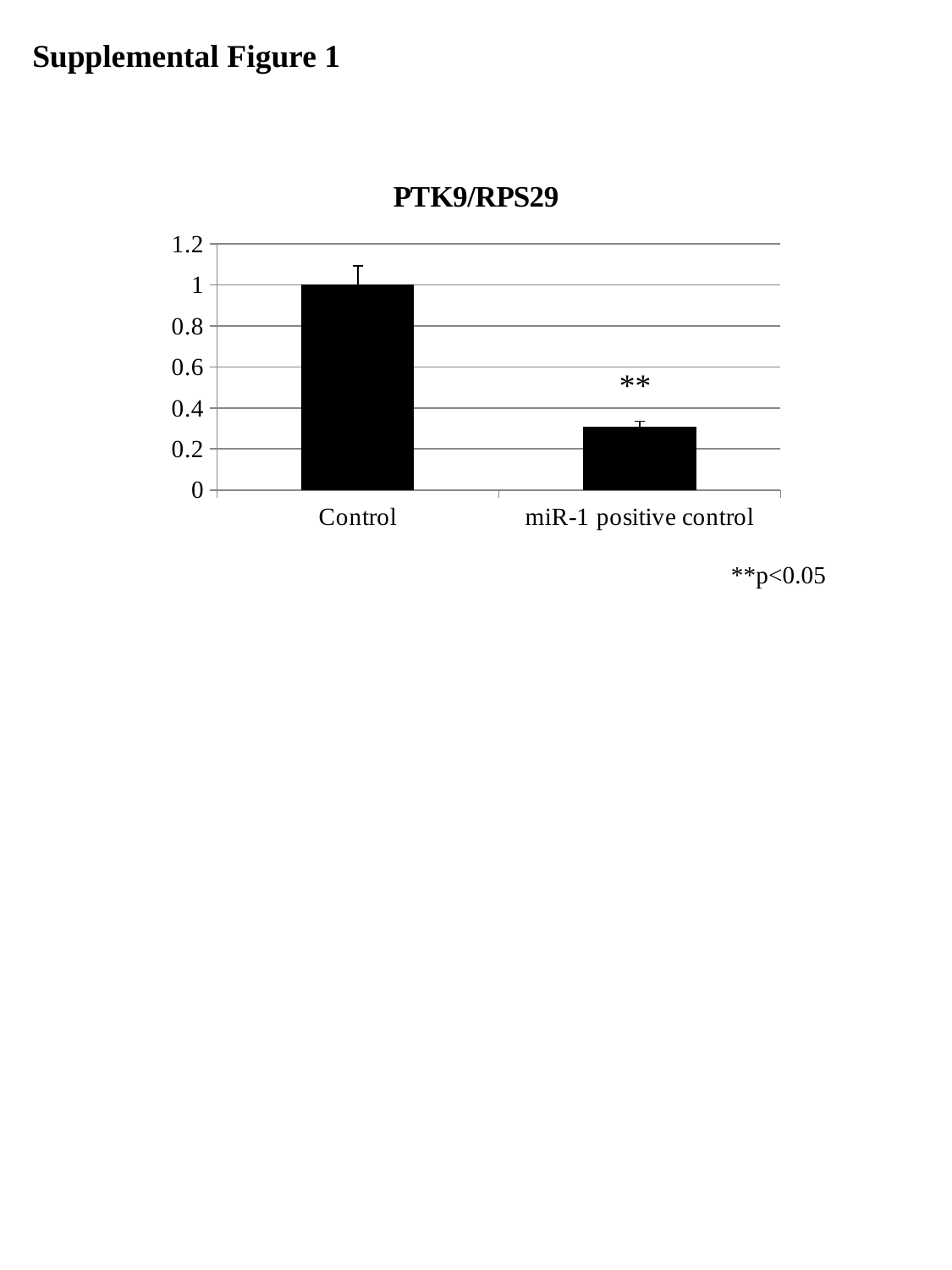

Supplemental Figure 1
### Chart:
| Category | PTK9/RPS29 |
|---|---|
| Control | 1.0 |
| miR-1 positive control | 0.309728667733576 |**
**p<0.05

Supplement: Additional file 1: Figure S1. — The efficacy of miRNA transfection was assessed by real time quantitative RT-PCR. miR-1 positive control transfection reduced its target gene (PTK9) expression significantly. RPS29 was used as a control housekeeping gene. [file 12860_2015_61_MOESM1_ESM.pptx]
