# Supplementary material for: miR-487b, miR-3963 and miR-6412 delay myogenic differentiation in mouse myoblast-derived C2C12 cells
Source: BMC Cell Biol. 2015 Apr 30;16:13. doi: 10.1186/s12860-015-0061-9 (PMC4433089; doi:10.1186/s12860-015-0061-9)
Supplement: Additional file 2: Figure S2. — The expression of miR-487b, miR-3963 and miR-6412 was assessed by RT-qPCR. The expression of these three miRNAs was significantly down-regulated in C2C12 cells cultured in differentiation medium, compared with cells cultured in proliferation medium. [file 12860_2015_61_MOESM2_ESM.pptx]

## Slide 1
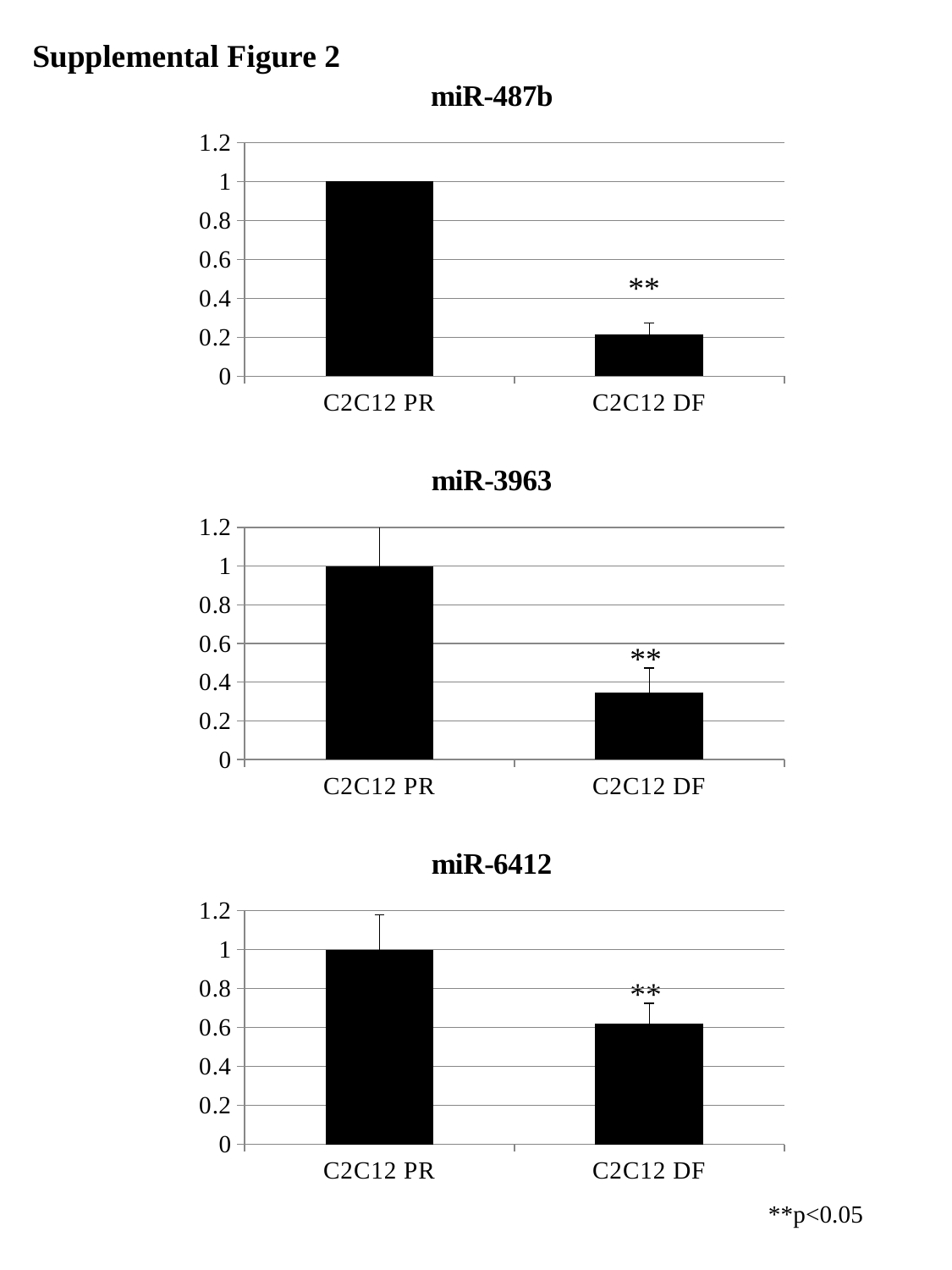

Supplemental Figure 2
### Chart:
| Category | miR-487b |
|---|---|
| C2C12 PR | 1.0 |
| C2C12 DF | 0.214620371283677 |**
### Chart:
| Category | miR-3963 |
|---|---|
| C2C12 PR | 1.0 |
| C2C12 DF | 0.344465724671624 |**
### Chart:
| Category | miR-6412 |
|---|---|
| C2C12 PR | 1.0 |
| C2C12 DF | 0.618224965524874 |**
**p<0.05
