# Supplementary material for: Erratum to: miR-487b, miR-3963 and miR-6412 delay myogenic differentiation in mouse myoblast-derived C2C12 cells
Source: BMC Cell Biol. 2016 Mar 10;17:9. doi: 10.1186/s12860-016-0083-y (PMC4830013; doi:10.1186/s12860-016-0083-y)
Supplement: Additional file 2: Figure S2. — (PPTX 62 kb) [file 12860_2016_83_MOESM2_ESM.pptx]

## Slide 1
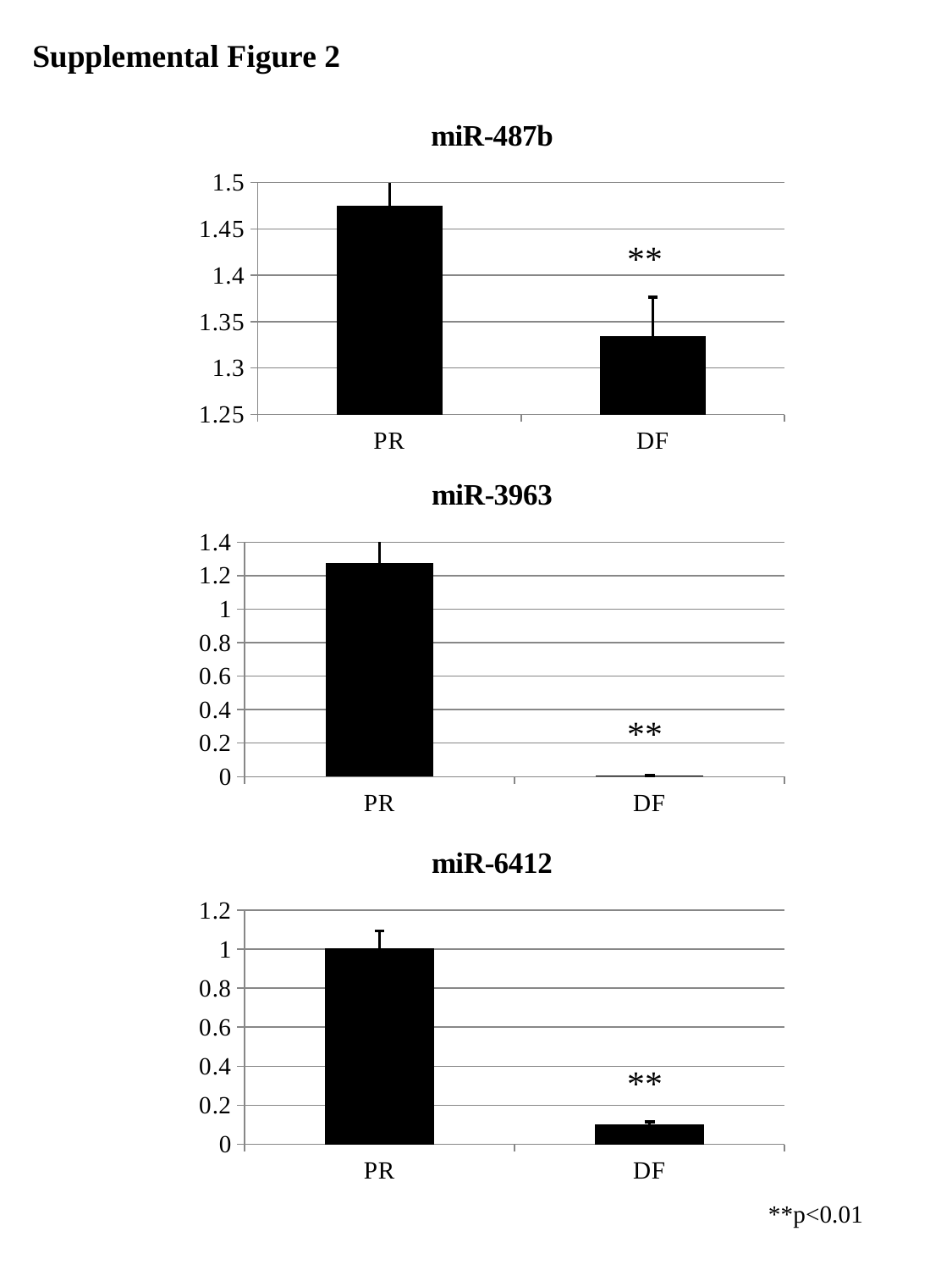

Supplemental Figure 2
### Chart:
| Category | miR-487b |
|---|---|
| PR | 1.4749388696946 |
| DF | 1.334096362863093 |**
### Chart:
| Category | miR-3963 |
|---|---|
| PR | 1.275478635967521 |
| DF | 0.00405085136860747 |**
### Chart:
| Category | miR-6412 |
|---|---|
| PR | 1.003644473572516 |
| DF | 0.1002305424671 |**
**p<0.01
